# Inter-breast Ratios of Tissue Dielectric Constant Values to Detect and Track Unilateral Breast Edema or Lymphedema

**DOI:** 10.7759/cureus.97657

**Published:** 2025-11-24

**Authors:** Harvey N Mayrovitz

**Affiliations:** 1 Medical Education, Nova Southeastern University Dr. Kiran C. Patel College of Allopathic Medicine, Davie, USA

**Keywords:** breast cancer, breast edema, breast lymphedema, breast measurements, breast tumor, edema threshold, tdc, tissue dielectric constant

## Abstract

Background: In the absence of sophisticated imaging, the assessment of breast edema or breast lymphedema relies mostly on patient-reported symptoms and subjective clinical assessments. These are useful, but they may occur late in the process, which limits the ability to detect them earlier. Measurements of breast tissue dielectric constant (TDC), due to its sensitivity to local tissue water, may provide a quantitative assessment method. The goal of this report is to present new results based on the analysis of inter-breast TDC measurements and the formulation of threshold ratios that may be useful as an aid in detecting and quantitatively tracking breast edema or lymphedema.

Methods: Breast TDC values obtained in healthy breasts of 165 women with a tumor in the other breast were used to determine the variability in healthy breast TDC values. In addition, in 78 of these women, in whom a subsequent biopsy showed them to have a benign tumor, inter-breast TDC ratios were determined (tumor breast/healthy breast). Edema thresholds were calculated based on the overall mean inter-breast ratio for these 78 women, to which a multiple of the overall standard deviation (SD) was added.

Results: The healthy breast TDC value for the 165 women was 28.74 ± 7.10, with a calculated coefficient of variation among patients of 24.7%. The inter-breast TDC ratio for the 78 women yielded an overall mean of 1.029 with a SD of 0.108. Based on this mean and variance, inter-breast edema thresholds corresponding to 2.0 SD, 2.5 SD, and 3.0 SD above the mean are 1.25, 1.30, and 1.35, respectively.

Conclusions: The inter-breast TDC ratios derived from bilateral breast TDC yield potentially useful inter-breast threshold values to help quantitatively assess the presence of breast edema or breast lymphedema. These proposed inter-breast threshold ratios ranged from 1.25 to 1.35, depending on the acceptable test sensitivity. The accuracy of these thresholds for assessing or tracking patients at risk of developing breast edema or lymphedema awaits future validation studies using quantitative imaging methods. The present values provide interim considerations for potential clinical use and serve as the requisite reference data for future validation studies.

## Introduction

More than a century ago, Archibald Leitch described the condition of peau d'orange [1]. This observation was prompted by the appearance of breast skin in patients with inflammatory breast cancer, which resembled the skin of an orange. The skin dimpling they noted may be attributable to lymphatic vessel obstruction by cancer cells, leading to heterogeneous dermal fluid accumulation and a dimpling appearance. Inflammatory breast cancer is relatively rare [2]. Its incidence ranges between 1.4 to 3.0 per 100,000 women and represents between 2% to 5% of all breast cancer diagnoses [3,4]. Some cases of breast edema are due to lymphatic blockage or even heart failure, but most cases of breast edema are related to breast cancer treatment [5]. Various assessments have been used, including skin thickness [6,7]. An early assessment was conducted on 74 patients treated with irradiation, some with and some without axillary lymph node dissection [8]. Most developed breast edema (26/33). However, breast edema was scored as mild, moderate, or severe based on differential breast size and the presence of peau d’orange. Although using breast size to assess breast edema is difficult [9], it has been reported that women with larger breasts are more likely to develop breast edema [10]. Another study of 208 women with breast cancer suggested that breast edema was related to two main variables: a greater BMI and the tumor located in the upper outer quadrant [11]. However, the presence of breast edema was judged on signs and symptoms that included pain, redness, and a feeling of heaviness. Questionnaires have also been used to subjectively assess breast edema [12]. In other studies, breast edema was diagnosed solely on clinical grounds [13,14].

Given the variability in diagnostic approaches, there is a need for a convenient, non-invasive quantitative assessment method [15-19]. One approach measures breast tissue dielectric constant (TDC). Previous work measured TDC values to a depth of 2.5 mm and suggested that an inter-breast TDC ratio of 1.4 or greater indicated breast edema. This threshold was based on the mean TDC value, to which 2 standard deviations (SD) were added [20,21]. To provide for a larger sample size, inter-breast TDC ratios were determined to the same measurement depth at a standard breast location in 61 women who were about to have a breast biopsy [22]. An inter-breast TDC ratio of 1.033 ± 0.099 was determined, yielding a 2.0 SD threshold of 1.23. Subsequent TDC measurements of women who were also awaiting a biopsy of a breast tumor yielded an inter-breast 2.0 SD TDC threshold ratio of 1.275 [23]. A potential confounding factor in these two threshold determinations is that they were based on comparing TDC values as ratios between the tumor-bearing and contralateral breasts, regardless of whether the tumor was subsequently determined to be benign or malignant [22,23]. Because the presence of a malignant tumor is likely to cause an abnormal elevation in TDC [24,25], a more clinically relevant inter-breast threshold ratio might, in retrospect, be determined without inclusion of breasts carrying malignant tumors. Thus, the goal of the present report is to provide composite TDC findings from analyses of healthy and tumor-bearing breasts and to establish initial inter-breast edema thresholds based solely on inter-breast TDC ratios in patients with benign tumors. 

## Materials and methods

Overview

Data obtained from two prior, original Institutional Review Board-approved studies (IRB, 2019-7-Non-NSU-Health and IRB-2021-307) were combined and then filtered to exclude data from patients diagnosed with a malignant breast tumor. The total number of patients in whom bilateral breast TDC measurements were made was 165, of whom 78 remained after filtering for inter-breast analysis. These 78 patients had a benign tumor in one breast and were used to determine the inter-breast ratio, which was calculated as the TDC value in the tumor-bearing breast divided by the TDC value in the healthy contralateral breast. TDC values were based on triplicate measurements in each breast, within the breast quadrant containing the tumor on the tumor-bearing breast, as well as on the anatomically similar site on the healthy breast. Potential breast edema threshold values were determined by computing the mean value for the inter-breast ratio and adding 2.0 SD, 2.5 SD, and 3.0 SD, which correspond to the inclusion of approximately 95.5%, 98.7%, and 99.7% of cases, respectively. The absolute TDC threshold was calculated based on values obtained only from healthy breasts of all patients (n = 165). Each TDC parameter was tested for normality using the Shapiro-Wilk statistic, which showed no significant departures from normality for these parameters. 

Subjects

Healthy breast data were from the non-affected breast of 165 patients who were awaiting a biopsy for a tumor in one breast. Inter-breast values were calculated from a subset of these patients (n = 78) diagnosed with a benign tumor. Table 1 summarizes the main features of these subjects, including tumor locations. The age range for the entire group was from 31 years to 86 years, with a mean ± SD of 63.2 ± 11.7 years. The BMI of the entire group ranged from 19.1 Kg/m² to 50.1 Kg/m² and averaged 29.1 ± 6.0 Kg/m². Thus, the group of patients included in this study was significantly overweight with a BMI value close to the threshold BMI that would be classed as obese (30.0 Kg/m^2^). Overall, the left breast had the largest percentage of tumors (58.2%), but for patients who had benign tumors, the tumor distribution was nearly equal, with 52.6% in the left breast and 47.4% in the right breast. More than two-thirds of all tumors were located in the upper two breast quadrants (69.7%), with the upper-outer quadrant bearing the largest percentage (46.1%).

**Table 1:** Subject demographics and tumor location The entries for age and body mass index (BMI) are the mean ± standard deviation. The entries for the other parameters are the number of patients and the percentages in parentheses. The tumor quadrants (A, B, C, and D) correspond to the breast locations upper inner, upper outer, lower inner, and lower outer, respectively. The healthy breasts from all patients were used to determine the absolute TDC threshold for breast edema. The data from patients with benign tumors was used to determine the inter-breast edema threshold.

			Tumor Breast		Tumor Quadrant			
	Age (Years)	BMI (Kg/m^2^)	Left	Right	A	B	C	D
All patients (N=165)	63.2 ± 11.7	29.1 ± 6.0	96 (58.2)	69 (41.8)	39 (23.6)	76 (46.1)	22 (13.3)	28 (17.0)
Patients with a benign tumor (N=78)	58.8 ± 12.3	29.4 ± 6.3	41 (52.6)	37 (47.4)	17 (21.8)	34 (43.6)	11 (14.1)	16 (20.5)

## Results

Healthy breast TDC values

**Figure 1:**
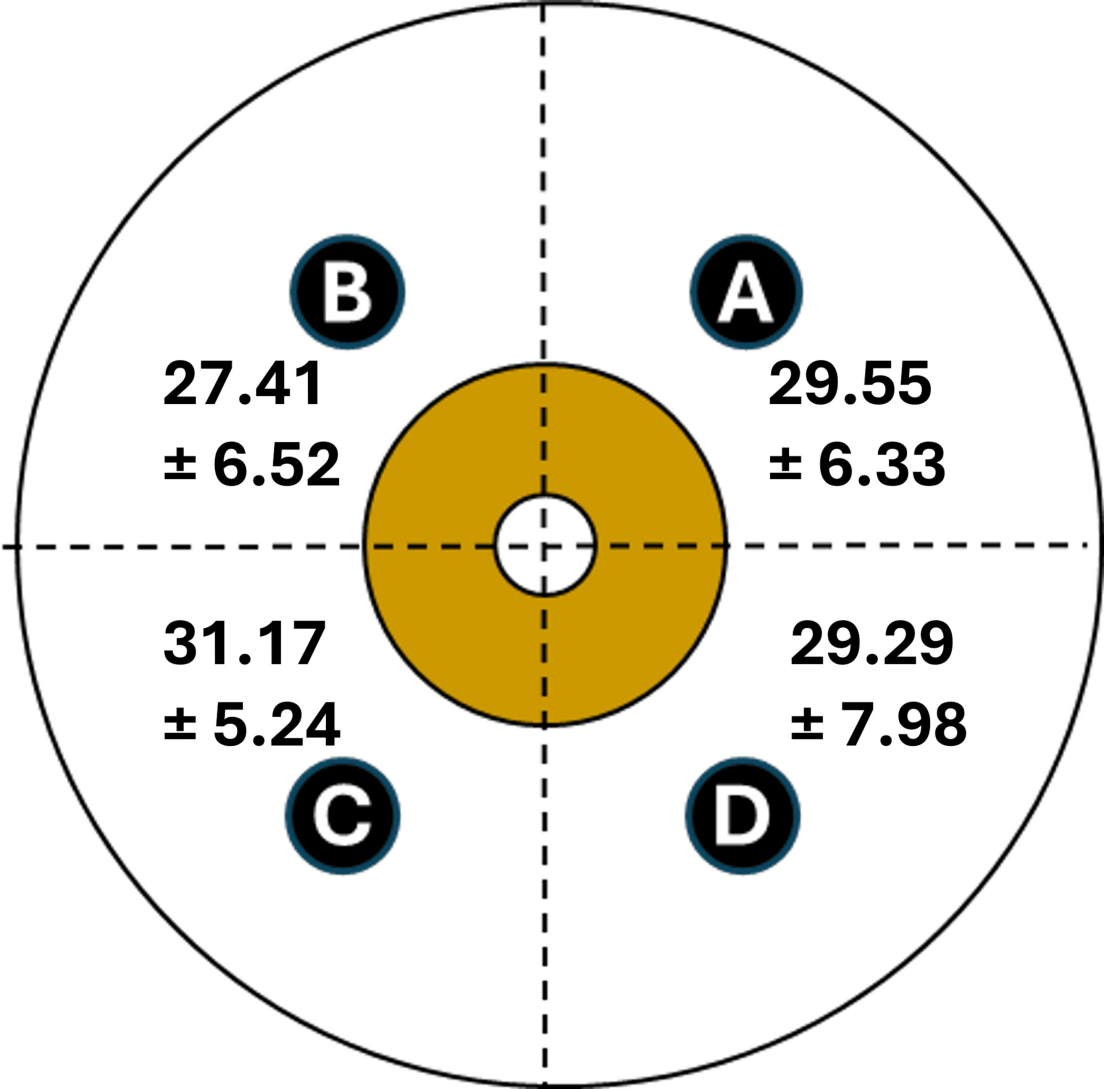
Tissue dielectric constant (TDC) values in healthy breasts TDC values ± standard deviation are shown for each quadrant of 165 healthy breasts. The quadrants A, B, C, and D correspond to upper inner, upper outer, lower outer, and lower inner quadrants. TDC values for quadrants A, B, C, and D are based on 39, 76, 22, and 28 triplicate measurement sets, respectively. There was no statistical difference in TDC values among quadrants.

Figure 1 illustrates the distribution of TDC values (mean ± SD) as determined in each breast quadrant for the healthy breast for all 165 patients. There was no significant difference among quadrant values based on analysis of variance (ANOVA) testing (p = 0.115). Averaging the quadrant TDC values for all 165 breasts yielded a healthy breast TDC value of 28.74 ± 7.10 with a calculated coefficient of variation among patients of 24.7% and a 2.0 SD threshold of 42.94.

Paired breast TDC values for benign tumor cases

**Figure 2:**
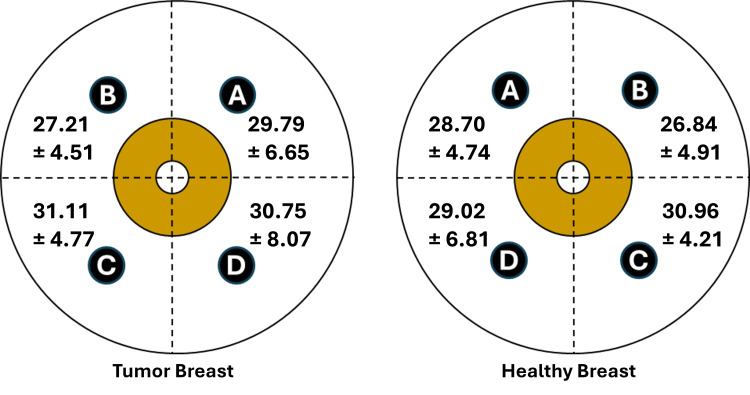
Inter-breast tissue dielectric constant (TDC) values for patients with a benign tumor TDC values ± standard deviation are shown for each quadrant of both breasts of 78 patients who bore a benign tumor in one breast. The quadrants A, B, C, and D correspond to upper inner, upper outer, lower outer, and lower inner quadrants. TDC values for quadrants A, B, C, and D are based on 17, 34, 11, and 16 triplicate measurement sets, respectively. There was no statistical difference in TDC values among quadrants for the tumor breast (p = 0.111) or the healthy breast (p = 0.128). Mean values of the tumor breast at each quadrant tended to be slightly greater than the healthy breast, but were not statistically significant at any quadrant.

Figure 2 illustrates the TDC values in both breasts for the 78 patients who had benign tumors. There was no significant difference among quadrant values based on ANOVA testing for the healthy breasts (p = 0.128) or the tumor-bearing breast (p = 0.111). Paired breast values for corresponding quadrants in the healthy and tumor-bearing breast were highly correlated (p < 0.001). Mean values of the tumor breast at each quadrant tended to be slightly greater than the healthy breast, but were not statistically significant at any quadrant, with p-values at all quadrants greater than 0.3. These quadrant differences were based on 17, 34, 11, and 16 triplicate measurement sets for quadrants A, B, C, and D, respectively. Combining all quadrant TDC values yielded a healthy breast average (N =78) of 28.26 ± 5.33 and a tumor-bearing breast average of 29.05 ± 6.03, p = 0.029. Thus, when considering the whole-breast average, independent of quadrant, there is a small inter-breast TDC difference (2.7%) in patients with a benign tumor.

Paired breast TDC values for malignant tumor cases

**Figure 3:**
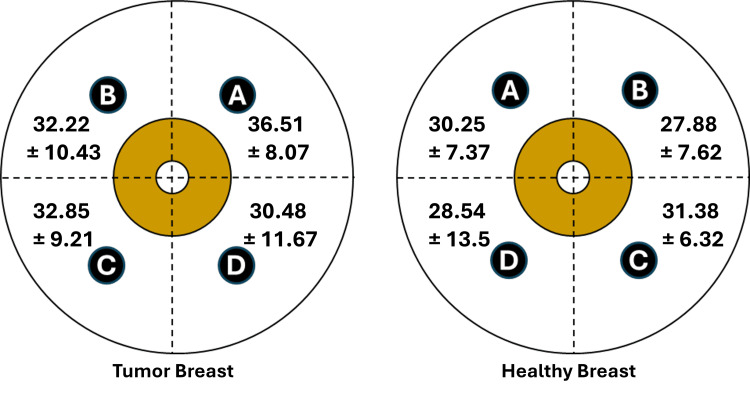
Inter-breast tissue dielectric constant (TDC) values for patients with a malignant tumor TDC values ± standard deviation are shown for each quadrant of both breasts of 87 patients who had a malignant tumor in one breast. The quadrants A, B, C, and D correspond to upper inner, upper outer, lower outer, and lower inner quadrants. TDC values for quadrants A, B, C, and D are based on 22, 42, 11, and 12 triplicate measurement sets, respectively. There was no statistical difference in TDC values among quadrants for the tumor breast (p = 0.303) or the healthy breast (p = 0.541). Mean values of the tumor breast at each quadrant tended to be greater than the healthy breast, but were statistically greater only in the upper quadrants, with p-values for quadrants A and B being < 0.001 and = 0.002, respectively.

Figure 3 illustrates the TDC values in both breasts for the 87 patients who had a malignant tumor. There was no statistical difference in TDC values among quadrants for the tumor breast (p = 0.303) or the healthy breast (p = 0.541). Paired breast values for corresponding quadrants in the healthy and tumor-bearing breast were highly correlated (p < 0.001). Mean values of the tumor breast at each quadrant tended to be greater than the healthy breast, but were statistically greater only in the upper quadrants, with p-values for quadrants A and B being < 0.001 and = 0.002, respectively. These quadrant differences were based on 22, 42, 11, and 12 triplicate measurement sets for quadrants A, B, C, and D, respectively. Combining all quadrant TDC values yielded a healthy breast average (N = 87) of 29.02 ± 9.84, and a tumor breast average of 33.17, p < 0.0001. Thus, when considering the whole-breast average, independent of quadrant, the tumor-bearing breast has a significantly greater TDC value (14.3%) than the contralateral healthy breast.

Inter-breast TDC ratios for benign tumor cases

The inter-breast ratio (tumor-bearing/healthy), based on the average breast TDC data from patients with benign tumors, was 1.029 ± 0.108. This value can be used to estimate inter-breast threshold ratios that, if exceeded, would suggest the presence of breast edema based on 2.0, 2.5, or 3.0 SD above this mean value. Table 2 summarizes these calculated values and practical thresholds. The 2.0 SD, 2.5 SD, and 3.0 SD thresholds would be estimated to include approximately 95.5%, 98.7% and 99.7% of cases, respectively. These estimates are based on breast averages determined by TDC measurements in each of the four quadrants. If measurements were to be made only in the upper quadrants, then estimated thresholds would be based on the 51 cases that were measured in the upper-inner and upper-outer quadrants. Depending on the assessment condition, these thresholds range from 1.25 to 1.38 as indicated in Table 2.

**Table 2:** Tissue dielectric constant (TDC) inter-breast ratios for breast edema Mean and SD for inter-breast ratios for 78 patients diagnosed with a benign tumor. Ratio determined as the average TDC value in the tumor-bearing breast/healthy breast based on TDC measurements in all four quadrants (N=78) or if measured only in upper quadrants (N=51). Thresholds were determined to be 2.0 SD, 2.5 SD, and 3.0 SD above the overall mean inter-breast ratio. The practical thresholds are rounded to two decimals from those calculated and are easier to remember.

Assessment Condition	Mean Inter-breast Ratio	Standard Deviation (SD)		Mean + 2.0 SD	Mean +2.5 SD	Mean +3.0 SD
Average of all quadrants TDC measurements (N =78)	1.029	0.108		1.245	1.299	1.353
	Practical Thresholds			1.25	1.30	1.35
Average of upper quadrants TDC measurements (N = 51)	1.026		0.117	1.260	1.318	1.378
	Practical Thresholds			1.26	1.32	1.38

## Discussion

The main new outcome of this study was the development of TDC parameters that could be useful for assessing breast edema or lymphedema. By examining the TDC values in 165 healthy breasts, the average value and its standard deviation allowed for the calculation of an absolute TDC threshold of 42.94. However, due to the large coefficient of variation in TDC values among these healthy breasts (24.7%), the findings suggest that establishing a practical breast edema threshold based on TDC measurements in a single breast is difficult, except possibly in cases of severe breast edema or lymphedema. Despite this deficiency, there is potential utility in making available the expected average breast TDC value and its SD among a large number of healthy breasts (28.74 ± 7.10). A prior study also measured TDC values in healthy breasts, but these were measured in women who had already received surgical treatment for cancer in their other breast [23]. They reported an average TDC value of 30.0 but did not report the SD among the 118 women in which this was measured. A subsequent report based on 65 women who were surgically treated for breast cancer and who had their non-affected breast measured prior to the initiation of radiotherapy had a healthy breast TDC value of 27.5 ± 5.0 [22].

In contrast to using TDC thresholds based on single-breast measurements, using the inter-breast TDC ratio as a parameter allows for the definition of threshold ratios that, if exceeded, suggest the presence of breast edema or lymphedema. These inter-breast threshold ratios, based on the analysis of TDC data from 78 patients, were 1.25, 1.30, and 1.35, corresponding to ratios of 2.0 SD, 2.5 SD, and 3.0 SD above the mean, respectively. The assessment of the sensitivity and specificity associated with these proposed thresholds depends on prospective quantitative evaluations in patients with breast edema or lymphedema, which are assessed by other means, such as with magnetic resonance imaging [26,27] or high-frequency ultrasound [13]. These studies have yet to be undertaken. However, some insight can be gained from a prior study in which breast edema was assessed clinically in such patients. An inter-breast ratio threshold of 1.4 was used to assess the impact of radiotherapy on breast edema during a two-year follow-up [22]. Using this threshold, which was based on measurements in only 15 healthy subjects, it was found that breast edema did not occur until three months after radiotherapy initiation. However, based on the multiple sequential inter-breast measurements reported in that study, but using the present threshold of 1.35, indicates that breast edema would have been detected as early as one week after radiotherapy. This differential conclusion highlights the potential importance of using the proper inter-breast TDC ratio, based on a reasonable sample size, as is the case in the present study.

Study limitations

One limitation is that the inter-breast threshold ratios derived from the present study have not been compared with diagnostic assessments in patients with confirmed breast edema or breast lymphedema. The threshold ratios represent predicted thresholds that are firmly based on statistical considerations but assume the sample of women evaluated is representative of those women in whom the thresholds will be prospectively applied. A second potential limitation is that the inter-breast ratios were determined in women who had a benign tumor in one of their breasts. However, the potential confounding effect of this is mitigated by two factors. Firstly, the breast TDC value was determined by averaging the TDC values of all breast quadrants, whereas the single tumor was present in only one quadrant. Secondly, the absolute TDC values measured in the tumor-bearing breast and the contralateral, unaffected breast differed on average by less than one TDC unit.

## Conclusions

Inter-breast TDC ratios derived from bilateral TDC values in 78 women who were free of malignant tumors yielded potentially useful inter-breast threshold values to help quantitatively assess the presence of breast edema or breast lymphedema. These proposed inter-breast threshold ratios ranged from 1.25 to 1.35, depending on the acceptable test sensitivity. The accuracy of these thresholds for assessing or tracking patients at risk of developing breast edema or lymphedema awaits future validation studies using quantitative imaging methods. The present values provide interim considerations for potential clinical use and serve as the requisite reference data for future validation studies. 
